# Health Technology Assessment of a new water quality monitoring technology: Impact of automation, digitalization and remoteness in dialysis units

**DOI:** 10.1371/journal.pone.0247450

**Published:** 2021-02-25

**Authors:** Borja García-Lorenzo, Carla Fernández-Barceló, Francisco Maduell, Laura Sampietro-Colom

**Affiliations:** 1 Assessment of Innovations and New Technologies Unit, Hospital Clínic Barcelona, University of Barcelona, Barcelona, Catalonia, Spain; 2 Department of Nephrology and Renal Transplantation, Hospital Clínic Barcelona, University of Barcelona, Barcelona, Catalonia, Spain; Shahrood University of Technology, ISLAMIC REPUBLIC OF IRAN

## Abstract

**Background:**

Water quality monitoring at the dialysis units (DU) is essential to ensure an appropriate dialysis fluid quality and guarantee an optimal and safe dialysis treatment to patients. This paper aims to evaluate the effectiveness, economic and organizational impact of automation, digitalization and remote water quality monitoring, through a New Water Technology (NWT) at a hospital DU to produce dialysis water, compared to a Conventional Water Technology (CWT).

**Methods:**

A before-and-after study was carried out at the Hospital Clínic Barcelona. Data on CWT was collected during 1-year (control) and 7-month for the NWT (case). Data on water quality, resource use and unit cost were retrospective and prospectively collected. A comparative effectiveness analysis on the compliance rate of quality water parameters with the international guidelines between the NWT and the CWT was conducted. This was followed by a cost-minimization analysis and an organizational impact from the hospital perspective. An extensive deterministic sensitivity analysis was also performed.

**Results:**

The NWT compared to the CWT showed no differences on effectiveness measured as the compliance rate on international requirements on water quality (100% vs. 100%), but the NWT yielded savings of 3,599 EUR/year compared to the CWT. The NWT offered more data accuracy (daily measures: 6 vs. 1 and missing data: 0 vs. 20 days/year), optimization of the DU employees’ workload (attendance to DU: 4 vs. 19 days/month) and workflow, through the remote and continuous monitoring, reliability of data and process regarding audits for quality control.

**Conclusions:**

While the compliance of international recommendations on continuous monitoring was performed with the CWT, the NWT was efficient compared to the CWT, mainly due to the travel time needed by the technical operator to attend the DU. These results were scalable to other economic contexts. Nonetheless, they should be taken with caution either when the NWT equipment/maintenance cost are largely increased, or the workforce involvement is diminished.

## Introduction

End-stage renal disease (ESRD) is the stage where the patient needs a renal replacement therapy (RRT) to survive, which include dialysis and/or renal transplant. The European Renal Association (ERA) reported almost 700,000 people needs RTT with an incidence of 81,000 people/year in Europe [1]. RRT patients experience a decrease in life expectancy and health-related quality of life. In Europe from the health system perspective in 2015, the mean cost per RRT dialysis patient/year ranged from 48,000 EUR (UK) to 111,000 EUR (Netherlands) [2]. The growing prevalence of patients reaching the Chronic Kidney Disease stage at which RRT is required, may even worsen this financial burden [3, 4].

Water quality monitoring at the dialysis units (DU) is essential to ensure an appropriate dialysis fluid quality and guarantee an optimal dialysis treatment [5]. With this purpose, the dialysis system is deployed within strict compliance with the current international standards (ISO-23500) [6] on quality management and standards for water purity. Two levels of water quality are defined: purified water and ultra-pure water [7]. Current scientific evidence shows benefits of ultrapure dialysis fluid for patients’ outcome [8–10]. Purified water is obtained at the water plant and afterwards ultra-pure water is obtained at the personal dialysis equipment, being ultra-pure water ultimately used to obtain the dialysis fluid. Therefore, a water quality control needs to be delivered at both the water plant and the personal dialysis equipment [11]. Guidelines regulate both purified and ultra-pure water quality [12].

This study is focused on the water quality monitoring technology at the water plant of a hospital DU to produce dialysis water, comparing the Conventional Water Technology (CWT) with a New Water Technology (NWT), which is claimed to have potential added value through automated, digitalized and remote monitoring. This paper aims to evaluate the effectiveness, and the economic and organizational impact of the NWT in the water quality monitoring at a DU, compared to the CWT, to support health funding decision-making [13]. There is a twofold novelty in this research, the first worldwide implementation of the NWT in clinical practice, and the performance of a Health Technology Assessment (HTA).

## Methods

### Technologies compared

The water quality monitoring is currently being carried out on a typical offline / manual approach by the CWT. Allowing for some differences across countries, the CWT quality monitoring consists of one manual daily measure on a paper checklist by a technical operator (TO), followed by manual digitalization of the measurements. Hardness, chlorine and conductivity parameters are measured manually with reactive kits. The Colony Forming Units (CFU) and Endotoxin Units (EU) are measured through microbiological cultures. The chemical elements analysis is carried out using the most appropriate determination technique for each element, being each one detailed in ISO-23500-3. A monthly meeting including the TO, the head of nursery of the DU and the head of the DU is usually conducted to verify the data and report it to the quality control supervisor. The quality control supervisor quarterly issues a quality report. S1 Table reports the technical comparisons between both technologies.

For the NWT, six daily measures are automatically and digitally recorded, instantly digitalized and sent to the data server. The TO can remotely access daily data from digital devices, having both detailed information as well as an overview of the quality parameters. Hardness, chlorine and conductivity parameters were measured through embedded sensors, while the CFU, EU and the chemical elements analysis were performed as for the CWT. The NWT integrates a system of warnings/alerts that TOs’ receives instantly if any parameter is near the threshold limit or out of standards, respectively (such as salt, water conductivity, etc.). The automated, digitalized and remote monitoring with the NWT allows for an optimization of the workflow claiming to reduce the TO workload; a higher data accuracy and reliability as well as a reduction in days with absence of data; and a more efficient review during the monthly meetings and quality act reports. Although potential network fails and under/over triggering of warnings/alarms can occur, the NWT contains three pillars to ensure a high reliability: physical products (medical products and non-medical products), services (product related services and non-product related services) and digital solutions. This structure allows for a validation during the manufacturing process, during the dialysis unit’s setup and an annual regular check on performance which leads to a high reliability over 98% (calculation based on uptime of the system). Table 1 summarises the main characteristics of the CWT and the NWT.

**Table 1:** Main characteristics of NWT vs. CWT.

CWT	NWT
1 daily measure	6 daily measures
Manual recording of data	Automatic and digital recording of data (potential network fails)
Measurements performed manually with reagents (potential human error, less accuracy)	Measurements performed automatically through embedded sensors (no human error, more accuracy)
Paper checklist with a need for posterior digitalization	Automatically digitalized report on all the water quality data
Connection with hospital network needed to access the data	Remote access to data from tablet
A discontinuous monitoring can lead to a late response on potential errors	Warning/alarm feature allows for preventive measures way before an error occurs (potential under/over triggering of warnings/alarms)
Requires TOs to physically attend the DU on a daily basis	TOs only need to attend the DU when salt or a preventive action is needed
Absence of daily data if an unexpected event happens and the TO cannot attend the DU	Automatically digitalized recording of data prevents any absence of daily data that might occur due to external circumstances
Manual chemical disinfections (4 times/year; quarterly)	Automated heat disinfections (52 times/year; weekly)

CWT: Conventional Water Technology; NWT: New Water Technology; TO: Technical Operator; DU: Dialysis Unit

Network fails: unfeasibility to send the water quality data by the NWT due to an error of the communication network (internet).

Warning: message alerting a parameter is close to the limits of its standard.

Alarm: message alerting a parameter is out of the limits of its standard.

### Study design

**Fig 1:**
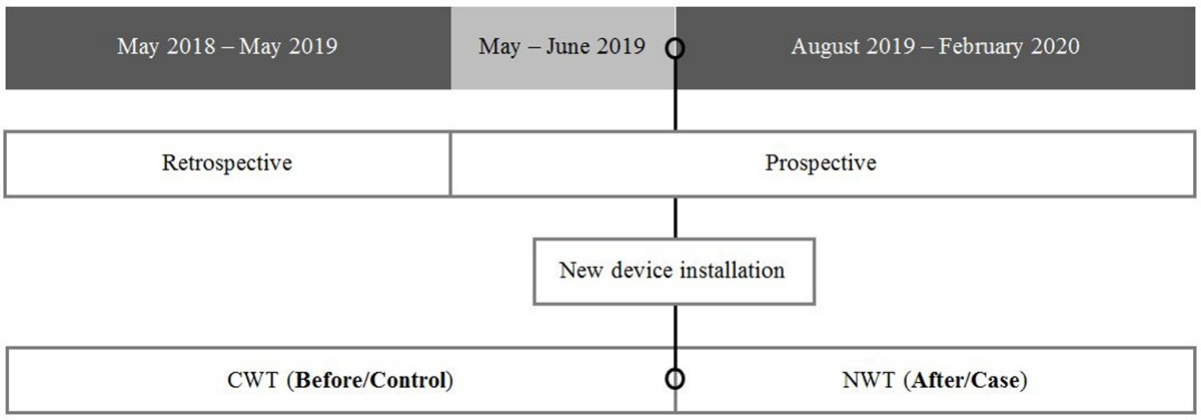
Study design. CWT: Conventional Water Technology; NWT: New Water Technology.

A before-and-after study was carried out (Fig 1). The CWT was operating during 1-year defined as control period (N = 313 observations/days; June-2018 –May-2019), while the NWT did so during 7-month defined as case period (N = 183 observations/days; August-2019 –February-2020). The 1-year control period contained 11-month retrospective data and 1-month prospective data (May-2019) to collect variables not available retrospectively. The 7-month case period was prospectively collected. The study of the NWT compared to the CWT was conducted in one of the satellite DUs attached to the Hospital Clínic Barcelona (HCB) (DU situated in a different physical location from HCB). This study did not include information on human/animal subjects or other materials from a natural setting requiring an approval by an ethics committee.

### Effectiveness and economic evaluation

Although the water plant is an important and mandatory element of the dialysis process, once the dialysis fluid has been formed, two *diasafe* filters to achieve ultra-pure dialysate, located in the dialysis machines, are mandatory for these treatments in which the dialysate itself is used as a replacement solution. With these safety filters, it is unlikely to improve clinical results depending on water quality and/or dialysate [14–16]. Therefore, differences in patient health outcomes were not assumed to be achieved by the NWT. Consequently, the water quality parameters, defined as maximum allowable contaminant levels [6, 17], were considered as effectiveness measure to conduct a cost-effectiveness analysis (CEA). Data on water quality parameters, resource use and unit cost (EUR 2019) were collected (daily, monthly and annual, as appropriate). Data was obtained from the review of quality control records, HCB administrative databases and Fresenius Medical Care (FMC). The list of collected variables is available on S2 Table.

A descriptive analysis (average/frequency and standard deviation) followed by statistical tests (t-test and chi-squared) on water quality and resource use variables comparing the CWT and the NWT was conducted. Neither sample size nor power calculation were performed since sample size of control and case groups were largely enough to compute the statistical tests.

Using annual data, effectiveness and costs were evaluated for each technology. The effectiveness was measured as the compliance rate of the water quality parameters with the recommended standards by the international guidelines [6]. The cost was computed as the annual aggregated cost, multiplying each resource use by its unit cost. Then, a CEA was planned and was summarized as the incremental cost-effectiveness ratio (ICER), defined as the incremental cost divided by the incremental effectiveness of the two competing alternatives (1).

$$ICER=\frac{C_{NWT}-C_{CWT}}{E_{NWT}-E_{CWT}}$$ (1)

ICER: Incremental Cost-Effectiveness Ratio; C_NWT_: Annual aggregated cost of the NWT; C_CWT_: Annual aggregated cost of the CWT; E_NWT_: Compliance rate of the water quality parameters of the NWT; E_CWT_: Compliance rate of the water quality parameters of the CWT

Since both technologies met the international guidelines [6] requirements on water quality, then no incremental effectiveness was found (see Table 2 in Results section). Therefore, according to the economic evaluation guidelines [18], a cost-minimization analysis (CMA) was conducted. The CMA was summarized as the incremental cost, defined as the difference between the annual aggregated cost of the two competing alternatives (2) [18].

$$CMA=C_{NWT}-C_{CWT}$$ (2)

CMA: Cost-Minimization Analysis; C_NWT_: Annual aggregated cost of the NWT; C_CWT_: Annual aggregated cost of the CWT

**Table 2:** Effectiveness measures. Water quality parameter standards, values and compliance rate.

Variable	Unit	Standard^f^	CWT		NWT		Difference NWT-CWT	
			Average (SD)	CR (%)	Average (SD) N = 183	CR (%)	Average	CR (p.p.)
							(p-value)	
			N = 313					
N° of daily measurements	Measures/day	n.a.	1 (0.00)	100	6 (0.00)	100	5 (n.a.)	0
Hardness	°fH	1	1 (0.00)	100	0,3 (0.02)	100	-0.7 (n.a.)	0
Chlorine	mg/l	0,1	0.1 (0.00)	100	0 (0.00)	100	-0.1 (n.a.)	0
Conductivity Meter (Inlet water)	μs/cm	n.a.	886 (185)	n.a.	1102 (87)	n.a.	216 (0.000)	n.a.
Conductivity Master (after 1^st^ RO)^a^	μs/cm	n.a.	15 (1)	n.a.	16 (4)	n.a.	1 (0.000)	n.a.
Conductivity Slave (after 2^nd^ RO)^b^	μs/cm	10	3.6 (0.47)	100	4.6 (0.41)	100	1 (0.000)	0
Rejection Rate^c^	%	n.a.	99.55 (0.11)	100	99.58 (0.02)	100	0.03 (0.001)	0
Colony Forming Units^d^ (CFU)	CFU/ml	100	1 (n.a.)	100	1 (n.a.)	100	0 (n.a.)	0
Endotoxin Units^e^ (EU)	EU/ml	0,25	0.005 (n.a.)	100	0.0624 (0.09)	100	0.0124 (n.a.)	0
Aluminium	mg/l	0,01	<0,01	100	<0,01	100	n.a.	0
Total Chlorine	mg/l	0,1	<0,1	100	<0,1	100	n.a.	0
Copper	mg/l	0,1	<0,01	100	<0,01	100	n.a.	0
Fluoride	mg/l	0,2	<0,2	100	<0,2	100	n.a.	0
Lead	mg/l	0,005	<0,005	100	<0,005	100	n.a.	0
Nitrate	mg/l	2	<0,5	100	<0,5	100	n.a.	0
Sulfate	mg/l	100	<2	100	<2	100	n.a.	0
Zinc	mg/l	0,1	<0,25	100	<0,25	100	n.a.	0
Calcium	mg/l	2	0.5375	100	<0,5	100	n.a.	0
Magnesium	mg/l	4	<0,1	100	0.104	100	n.a.	0
Potassium	mg/l	8	<0,5	100	<0,5	100	n.a.	0
Sodium	mg/l	70	0.5398	100	<0,5	100	n.a.	0
Antimony	mg/l	0,006	<0,001	100	<0,001	100	n.a.	0
Arsenic	mg/l	0,005	<0,002	100	<0,002	100	n.a.	0
Barium	mg/l	0,1	<0,01	100	<0,01	100	n.a.	0
Beryllium	mg/l	0,0004	<0,0001	100	<0,0001	100	n.a.	0
Cadmium	mg/l	0,001	<0,001	100	<0,001	100	n.a.	0
Chromium	mg/l	0,014	<0,005	100	<0,005	100	n.a.	0
Mercury	mg/l	0,0002	<0,0001	100	<0,0001	100	n.a.	0
Selenium	mg/l	0,09	<0,005	100	<0,005	100	n.a.	0
Silver	mg/l	0,005	<0,005	100	<0,005	100	n.a.	0
Thalium	mg/l	0,002	<0,001	100	<0,001	100	n.a.	0

CR: compliance rate with ISO standards; N: number of measurements; RO: Reverse Osmosis; p.p.: percentage point; n.a.: non-applicable (due to one single data or variable measured as a threshold i.e. data reported as “<0.1”); SD: standard deviation

^a^ Control: N = 29

^b^ SEN standards were assumed due to lack of ISO standards.

^c^ Rejection rate: ((Conductivity Meter- Conductivity Slave)/ Conductivity Meter)·100

^d^ Control: N = 24; Case: N = 7

^e^ Control: N = 2; Case: N = 2

^f^ISO-13959

Prospectively collected resource use that was not available for a whole year, and the subset that was not monthly collected (based on information retrieved from the HCB/FMC), were annually extrapolated assuming linearity. Besides, the working time of the DU employees was based on the opportunity cost, which assumed that the working time freed due to the NWT improvements may be spent in any other efficient task at DU, i.e. a 100% productivity of the DU employees was assumed. Therefore, even though the figures from human resources did not have a monetary impact to the hospital budget, they were accounted in economic terms and included in the total cost of each alternative. This analysis took a hospital perspective considering only direct costs incurred by HCB. Costs were not discounted due to the short time frame of the analysis (1-year).

To assess robustness and transferability of the CMA results according to different guidelines’ requirements, health systems and economic contexts, extensive deterministic and scenarios sensitivity analyses were performed.

A two-way sensitivity analysis on material resource use was performed. There was no consumption of supplementary task time related to the CWT and no filter replacements on the NWT. Given that these two figures were unlikely to occur, and therefore, an annual extrapolation would have been biased, the same level of consumption of these parameters for both technologies was assumed.

Scenario 1 simulated a twofold improvement in the efficiency of water consumption. On the one hand, a water leak occurred during the NWT period, which was adjusted to the monthly average consumption. On the other hand, the water consumption of the NWT was extrapolated using only the period after an improvement in the water setting on the NWT (December-2019 to February-2020).

Scenario 2a and 2b were built to offer different economic contexts where material and human resource unit cost may vary (scenario 2a-2b lower and higher unit costs were assumed respectively).

Scenarios 3a to 3e simulated five scenarios that simultaneously varied the technology cost, its maintenance cost and its lifecycle, proposing a conservative, an intermediate and three favourable scenarios for the NWT.

Since most of the DUs world-wide are composed by either a single DU or several DUs in the same physical location, different from this study, scenario 4 assumed that either DUs attached to the healthcare centre were in the same location or the healthcare centre had a single DU. Therefore, no TO travel time for both CWT and NWT was assumed.

To simulate the compliance of international recommendations on continuous monitoring [6], scenario 5 was built where three measures per day instead of one needed to be performed.

Finally, scenario 6 relaxed the assumption on the full-productivity of the DU employees reducing their productivity from 100% to 70%.

### Organizational assessment

Data was gathered from the database on resource use and technical variables (TO attendance, network fails on sending water quality data, etc.), from an online survey on employees’ satisfaction, workflow and workload. Respondents’ inclusion criterion was those people involved in the water quality control at the DU (i.e. the head, the quality coordinator, the nursery coordinator and the TOs). Respondents were contacted by email on May 2019, and to avoid bias according to the learning process of the NWT use, the same survey was answered online by the respondents on October 2019 and on January 2020, after the NWT installation. The study was conducted in a single DU and therefore the sample size was rather low (N = 5). Nonetheless, no other people were involved in the water quality monitoring at this DU. The survey contained a written informed consent and 11 questions comparing the NWT to the CWT in terms of time management, reliability of data collected and assessment of positive and negative aspects. Three questions formats were used: Likert scale, ranking and open questions. The survey is available in S1 Survey.

A descriptive analysis on the technical variables comparing both technologies was performed. Then, a descriptive analysis of the two survey answers was also conducted. Consistency and major differences between the two surveys were analysed.

## Results

### Effectiveness and economic evaluation

Although statistical significant differences on the water quality parameters between the NWT and the CWT were found, they were not considered technically relevant for the water quality since parameters for both technologies complied with the international guideline standards [6] (NWT: 100% vs. CWT: 100%). Table 2 shows the statistics of water quality parameters and the compliance rates for each technology.

CMA results showed that the NWT was associated to overall lower cost compared to the CWT (-3,599 EUR/year). These savings were mainly caused by a significant decrease in the human (-7,101 EUR/year) and the material resource use (-2,892 EUR/year mainly in salt and water consumption, and filter replacements). As expected, these savings were offset with the incremental cost of the NWT equipment/maintenance compared to those of the CWT (6,394 EUR/year). Table 3 shows the monthly average and annual aggregated resource use, and Table 4 shows monthly average and annual aggregated cost.

**Table 3:** Monthly average and annual aggregated resource use.

	Monthly average			Annual aggregated			
Variables (unit)	CWT (SD)	NWT (SD)	Incremental (NWT-CWT)	CWT	NWT	Incremental (NWT-CWT)	Unit Cost ^f^ (min-max) (EUR)
**Human Resources (hours)**							
TO daily total time	39 (n.a.)	8 (1.5)	-31	486	92	-394	-
*O travel time*	28 (n.a.)	2 (0.3)	-26	338	28	-310	-
*TO routine time*^*a*^	6 (n.a.)	3 (0.7)	-3	68	35	-33	-
*TO data management time*	7 (n.a.)	2 (0.1)	-5	79	26	-53	-
*TO supplementary task time*^*a*^	0 (n.a)	17 (45)	17	0^c^	3^b^	3	-
Monthly meeting time^a^	30 (n.a.)	20 (0)	-10	6	4	-2	-
Water plant disinfection time^e^	n.a.	n.a.	n.a.	8	0	-8	-
Water plant calibration time	n.a.	n.a.	n.a.	0	1	1	-
Revalidation time	n.a.	n.a.	n.a.	7	7	0	-
**Material**							
Culture	1 (0)	1 (0)	0	12	12	0	83
Salt (kg.)	775 (n.a.)	368 (49)	-407	9300	4414	-4886	0.27
Supp. task material^d^	n.a.	n.a.	n.a.	1^c^	0^b^	-1	n.a.^d^
Water consumption (litres)	426960 (62935)	397714 (43346)	-29245	5123516	4772571	-350945	0.0013–0.0016
*Water consumption (litres/treatment)*	459 (64)	353 (4)	-106	n.a.	n.a.	n.a.	0.0013–0.0016
Electricity consumption (KwH)	3014(334)	3292 (425)	278	36163	39500	3337	0.0918–0.0921
Reagent (ml)	18 (n.a.)	0 (0)	-18	226	0	-226	0.71–2.31
Filter replacement	n.a.	n.a.	n.a.	24	4	-20	24.4–29.5
Water plant disinfection (litres)^e^	n.a.	n.a.	n.a.	30	0	-30	5
Water plant calibration (kit)	n.a.	n.a.	n.a.	0	1	1	25–35
**Technology**							
CWT equipment	n.a.	n.a.	n.a.	1	0	n.a.	66500
Maintenance CWT (fee)	n.a.	n.a.	n.a.	1	0	n.a.	7382
NWT equipment	n.a.	n.a.	n.a.	0	1	n.a.	111150
Maintenance NWT (fee)	n.a.	n.a.	n.a.	0	1	n.a.	9311

CWT: Conventional Water Technology; NWT: New Water Technology; TO: technical operator; n.a.: non-applicable; SD: standard deviation; -: non-available

^a^ Monthly average has been measured as minutes/month.

^b^ There is supplementary task time but no material since the corrective action did not require any material for it to be performed.

^c^ There is supplementary task material but no time since the corrective action was detected and fixed during the daily routine time

^d^ A wide range of materials are used in the supplementary tasks. The list is available upon request to authors.

^e^ All disinfections performed have been preventive.

^f^ Unit costs of human resources are available upon request to authors.

**Table 4:** Monthly average and annual aggregated cost.

	Monthly average (EUR)			Annual aggregated (EUR)		
	CWT (SD)	NWT (SD)	Incremental (NWT-CWT)	CWT	NWT	Incremental (NWT-CWT)
**Human Resources**						
TO daily total time	691 (n.a.)	114 (40)	-577	8297	1371	-6926
*TO travel time*	493 (n.a.)	34 (8)	-460	5918	402	-5516
*TO routine time*	100 (n.a.)	44 (18)	-56	1198	526	-672
*TO data management time*	99 (n.a.)	32 (9)	-67	1182	384	-798
*TO supplementary task time*	0 (n.a.)	5 (13)*	5	0^b^	59^a^	59
Monthly meeting time	43 (n.a.)	28 (0)	-15	521	347	-174
Water plant disinfection time^c^	41 (0)	0 (0)	-41	493	0	-493
Water plant calibration time^c^	0 (0)	1.6 (0)	1.6	0	19	19
Revalidation time	11 (n.a.)	11 (n.a.)	0	134	134	0
Total	735	143	-592	8819	1718	-7101
**Material**						
Culture	83 (0)	83 (0)	0	996	996	0
Salt	209 (n.a.)	99 (13)	-110	2511	1192	-1319
Supp. task material	0,5 (n.a.)	0 (0)	-0,5	5^b^	0^a^	-5
Water consumption	655 (120)	593 (91)	-62	7858	7114	-744
*EUR/treatment*	0,71 (0,12)	0,49 (0)	-0,22	n.a.	n.a.	n.a.
Electricity consumption	277 (256)	302 (294)	25	3330	3625	295
Reagent	32 (n.a.)	0 (0)	-32	382	0	-382
Filter replacement	54 n.a.)	0 (0)	-54	647	0	-647
Water disinfection	13 (n.a.)	0 (n.a.)	-13	150	0	-150
Water calibration	0 (n.a.)	5 (n.a.)	5	0	60	60
Total	1324	1082	-241	15879	12987	-2892
**Technology**						
Equipment^d^	554	926	372	6650	11115	4465
Maintenance	615	776	161	7382	9311	1929
Total	1169	1702	533	14032	20426	6394
**Total incremental cost**	3228	2928	-300	38730	35131	-3599

CWT: Conventional Water Technology; NWT: New Water Technology; TO: technical operator; n.a.: non-applicable; SD: standard deviation

^a^There is supplementary task time but no material since the corrective action did not require any material for it to be performed.

^b^There is supplementary task material but no time since the corrective action was detected and fixed during the daily routine time

^c^ Based on the hospital perspective the cost of this resource use has not been included in total cost.

^d^The annual NWT and CWT cost were calculated based on a 10-year lifecycle.

The two-way sensitivity analysis and all scenarios except Scenario 3a and 4, showed savings ranging up to -21,218 EUR/year, where three measures per day instead of one needed to be performed, therefore presenting the NWT as an efficient technology compared to the CWT. In Scenario 3a, where conservative technology cost, its maintenance cost and its lifecycle for the NWT were considered the NWT did not yield savings, showing a low incremental cost (487 EUR/year). In Scenario 4, when either satellite DUs were in the same location or the healthcare centre had a single DU, the NWT did not longer yield savings showing an incremental cost of 1,918 EUR/year. Table 5 shows the results of the deterministic sensitivity analysis.

**Table 5:** CMA and deterministic sensitivity analysis results.

Type of sensitivity analysis	Parameters: Base case value	Parameters: New value	Incremental cost (EUR)
Base Case			- 3598
Bivariant analysis	Supplementary task time under CWT = 0 minutes	Supplementary task time under CWT = 206 minutes	-3550
	Filter replacement under NWT = 0 filter replacement	Filter replacement under NWT = 1 filter replacement	
**Scenarios**			
Scenario 1	Water consumption: 4772571 litres	Water consumption: 4122000 litres	-5003
Scenario 2a	Material and human resource unit cost under base case	Lower material and human resource unit cost (EUR, -20%)	-1467
Scenario 2b		Higher material and human resource unit cost (EUR, +20%)	-5398
Scenario 3a	Equipment: 111150 EUR Maintenance: 9311 EUR Life cycle: 10 years	Equipment: 133380 EUR Maintenance: 11173 EUR Life cycle: 10 years	487
Scenario 3b		Equipment: 133380 EUR Maintenance: 11173 EUR Life cycle: 13 years	-2591
Scenario 3c		Equipment: 111150 EUR Maintenance: 9311 EUR Life cycle: 13 years	-6163
Scenario 3d		Equipment: 88920 EUR Maintenance: 7449 EUR Life cycle: 10 years	-7684
Scenario 3e		Equipment: 88920 EUR Maintenance: 7449 EUR Life cycle: 13 years	-9736
Scenario 4	Travel time under CWT: 338 hours Travel time under NWT: 28 hours	CWT Travel time: 0 hours NWT Travel time: 0 hours	1918
Scenario 5	Resource use of CWT under base case	Three times the resource use of CWT under base case	-21218
Scenario 6	TO daily total time savings: 7000 EUR	TO daily total time savings: 5000 EUR	-1468

CWT: Conventional Water Technology; NWT: New Water Technology; TO: Technical Operator

### Organizational assessment

Improvements in terms of the DU employees’ workflow and TO workload were observed. Network fails reported did not affect the data collection, but they affected the real-time data transmission and access. No Information Technology (IT) errors occurred during the study. Additionally, warnings related to the salt amount control were already expected since they were aimed at optimizing salt consumption. No alarms were reported. The NWT allows for 6 daily measurements, which offers more data reliability along with the automation and digitalization of water quality parameters reports. This led to guaranteeing daily availability of water quality information (missed information: 20 days/year for CWT vs. 0 days/year for NWT) and a significant reduction of TOs’ attendance to the DU. Particularly, the main TO has reduced the average visits by 80% (NWT: 4 days/month 4 vs. CWT:19 days/month). Furthermore, the Saturday TO was no longer needed. Tables 6 and 7 show the demographic characteristics of the respondents and the results of the TO workload and technical variables, respectively.

**Table 6:** Demographic characteristics of respondents.

Variable	Mean / percentage (SD/ frequency)
Age (years)	46 (10)
Sex (female)	40 (2)
Position at the Dialysis Unit	
Head	20 (1)
Quality Control Coordinator	20 (1)
Nursery Coordinator	20 (1)
Technical Operator	40 (2)

SD: Standard deviation

**Table 7:** Organizational assessment results: TO workload and technical variables.

Variable	Monthly average (SD)			Annual Aggregated		
	CWT	NWT	Difference (NWT-CWT)	CWT	NWT	Difference (NWT-CWT)
Workload						
Main TO attendance to DU	19 (5)	4 (1)	-16	233	57	-176
Saturday TO attendance to DU	4 (1)	0 (0)	-4	45	0	-45
Holidays TO attendance to DU^a^	n.a.	n.a.	n.a.	18	5	-13
Technical						
Missing data days	1.6 (1.2)	0 (0)	-1.6	20	0	-20
Network fails	n.a.	1 (2)	n.a.	n.a.	9	n.a.
Warnings	n.a.	5 (0.5)	n.a.	n.a.	55	n.a.
Alarms	n.a.	0 (0)	n.a.	n.a.	0	n.a.

TO: technical operator; DU: Dialysis Unit; n.a.: non-applicable; SD: standard deviation

Network fails: unfeasibility to send the water quality data by the NWT due to an error of the communication network (internet).

Warning: message alerting a parameter is close to the limits of its standard.

Alarm: message alerting a parameter is out of the limits of its standard.

^a^ Since the holidays TO attendances was not regular, monthly averages were not computed

Misunderstandings of the data reported by the NWT were found in the first survey. Therefore, training was provided and, on the second survey no issues in understanding were reported anymore. Results of the second survey are reported in S1 Fig. shows the Likert scale questions’ complete results.

All respondents were globally more satisfied with the NWT compared to the CWT. Likert scale questions showed that remote and continuous monitoring, reliability of data and process regarding audits for quality control were better valued for NWT than for CWT. With the NWT, daily workload was significantly improved for the two TOs and remained the same for the rest of the employees; while monthly meeting dynamism remained the same for two-third of the respondents. Respondents answered as first and second most important benefits of the NWT the data reliability and higher control due to higher frequency of monitoring. Regarding the most pressing issues of the NWT potential network failures/no data reception and potential IT errors related to the NWT (e.g. false alarms, broken hardware) were ranked in the first and second place.

Answers from open questions pointed out that the NWT offered the opportunity to easily collect both certain water quality parameters (temperature, salt reduction, membrane yield) and the traceability of the preventive/corrective actions, which the CWT did not offer. Survey results showed improvements depending on the employees’ position (survey results available upon request to authors).

## Discussion

The NWT compared to the CWT provides with automation, digitalization and remote water quality monitoring for the first time worldwide in the clinical practice, performing an HTA. The HTA performed contains the three main pillars to health funding decision-making: evidence-based effectiveness, economic and organizational assessments, accordingly to the European Renal Best Practice Guidelines [19], to provide with value-based health care [13]. Accordingly, this study has shown that the NWT compared to the CWT is equally effective (water quality), cost-saving from the hospital perspective, and shows improvements in organizational aspects in terms of the data accuracy and reliability and, DU employees’ workflow, workload and satisfaction.

CMA results were robust to the linearity and full-productivity assumptions; however they were sensitive to the NWT’s equipment/maintenance cost and its lifecycle, having or not satellite DUs, and stricter quality control standards framework Neither the saving of the base case nor the incremental cost of an increase of the NWT equipment/maintenance cost, represent a significant impact on the hospital DU’s budget. However, either the decrease of the NWT equipment/maintenance cost or a stricter quality control standards framework, would yield significant savings for the hospital DU’s budget.

The continuous monitoring would also decrease the risk assessment related to undetected water contaminants. This fact is especially relevant for heavy equipment such as the water plant, which is not easy to be replaced, because it may lead to severe consequences, such as dialysis treatment delays. This saving would be relevant for a hospital DU’s budget, and would still comply with the higher standard requirements.

This study is not free of limitations. The first is related to the difference in the number of observations between the control and case period, and the consequently linearity assumption of resource use variables. On the one hand, the low variability of retrospective data on quality water parameters allowed us to use 7 months of prospective data for the NWT as a representative annual sample. On the other hand, due to both the expected low monthly variability and the lack of a seasonality pattern of most of resource use variables, the linearity assumption was fair to compute annual figures. However, this assumption may not apply for two variables, the time spent on the supplementary tasks of the CWT and the filter replacement of the NWT. To test the robustness of the CMA, a two-way sensitivity analysis varying both resource use was conducted. The results showed that none variable had a significant impact on the results. Another weakness may be the unlikeliness that 100% productivity of the DU employees is met. To test this assumption, a sensitivity analysis varying the productivity to 70% was assumed, showing that the NWT still yielded savings. Other limitations are related to the sample size of the satisfaction and organisation impact survey.

The automation, digitalization and remote monitoring of the NWT may provide potential benefits. Since HCB is a reference healthcare centre in the management of dialysis process using *diasafe*, potential patient outcomes may be observed in other healthcare centres, especially when considering interruption of treatment. Furthermore, TOs at HCB are trained by FMC providing with a better understanding and management of the CWT than in centres where TOs do not receive this training. Therefore, the advantages of the NWT may be larger in the latter centres. From the economic perspective, the NWT benefits may provide economies of scale for healthcare centres with several satellite DUs, and significantly increase savings when complying the international recommendations. Further research is encouraged in other clinical practice contexts to bring light on these assumptions.

To conclude, the NWT guaranteed the standards compliance [6, 17] of the water quality monitoring and, compared to the CWT, yielded savings from the hospital perspective. Furthermore, the NWT compared to the CWT offered more data accuracy and reliability, and optimized the DU employees’ workflow and TO’s workload. Therefore, the NWT is an efficient equipment compared to the CWT as water quality monitoring technology at DU. These results were robust to the assumption considered and scalable to other economic contexts. Nonetheless, they should be taken with caution either when the NWT equipment/maintenance cost are largely increased, or no travel time is needed to attend the DU. While the compliance of international recommendations on continuous monitoring was performed with the CWT, the online monitoring of the NWT would allow to largely increase its efficiency, and consequently the above cautions may be relaxed.

## Supporting information

S1 Table Devices comparison between CWT and NWT. CWT: Conventional Water Technology; NWT: New Water Technology.
(DOCX)

S2 Table List of collected variables. HCB: Hospital Clínic de Barcelona; FMC: Fresenius Medical Care; TO: Technical Operator; DU: dialysis unit; RO: Reverse Osmosis; n.a.: non-applicable.
(DOCX)

S1 Survey (DOCX)

S1 Fig Likert scale questions’ results. (DOCX)
