# Supplementary material for: Health Technology Assessment of a new water quality monitoring technology: Impact of automation, digitalization and remoteness in dialysis units
Source: PLoS One. 2021 Feb 25;16(2):e0247450. doi: 10.1371/journal.pone.0247450 (PMC7906308; doi:10.1371/journal.pone.0247450)
Supplement: S1 Table — CWT: Conventional Water Technology; NWT: New Water Technology. (DOCX) [file pone.0247450.s001.docx]

**S1 Table. Devices comparison between CWT and NWT.**

| **CWT** | | | **NWT** | |
| --- | --- | --- | --- | --- |
| **Device Name** | **Role / Purpose** |  | **Device Name** | **Role / Purpose** |
| **AquaBDUO 1500** | **Double Stage Reverse Osmosis System** |  | **AquaBPlus** | **Double Stage Reverse Osmosis System and Heat Disinfection Module.** |
|  |  |  | AquaBplus 2000 | First Stage RO System |
|  |  |  | AquaBplus B2 1500 | Second Stage RO System |
|  |  |  | AquaBplus HF | Heat Disinfection Module |
|  |  |  | DataCOM with Housing | Data Communication System |
|  |  |  | Cellular Module for DataCOM | Data Communication System Cellular Module |
| **Not applicable** | **Offline monitoring checklist** |  | **AquaSENS** | **Pretreatment Online Monitoring System** |
|  |  |  | AquaSens Control System Advanced | Pretreatment Online Monitoring Control System |
|  |  |  | AquaSens Total Chlorine Monitoring | Total Chlorine Monitoring Option |
|  |  |  | AquaSOFT | Hardness Monitoring Option |
|  |  |  | AquaSens BrineTankMonitoring E80,150,225 | Brine Tank Monitoring Option |
|  |  |  | AquaSens Media Filter / Autotrol | Backwash Monitoring Option |
|  |  |  | AquaSens Pressure Monitoring | Pressure Monitoring Option |
|  |  |  | AquaSens Electr WaterConsumption Counter | Electronic Water Consumption Counter |
|  |  |  | DataCOM with Housing | Data Communication System |
|  |  |  | Cellular Module for DataCOM | Data Communication System Cellular Module |
| **Not applicable** | **Offline Documentation System** |  | **IDMS – Infrastructure Data Management System** | **Web based platform for** |
|  | Validation folder (IQ, OQ, PQ & Mo) |  |  | Automatic, centralized and consolidated documentation according to ISO 23500 |
|  | Maintenance plan |  |  |  |
|  | Dialysis Fluid Sample plan |  |  |  |
|  | Dialysis Fluid Quality results |  |  |  |
|  | Disinfection plan and records |  |  |  |
|  | Setup records |  |  |  |
|  | Offline monitoring control checklists |  |  |  |

CWT: Conventional Water Technology; NWT: New Water Technology
