# Supplementary material for: Health Technology Assessment of a new water quality monitoring technology: Impact of automation, digitalization and remoteness in dialysis units
Source: PLoS One. 2021 Feb 25;16(2):e0247450. doi: 10.1371/journal.pone.0247450 (PMC7906308; doi:10.1371/journal.pone.0247450)
Supplement: S2 Table — HCB: Hospital Clínic de Barcelona; FMC: Fresenius Medical Care; TO: Technical Operator; DU: dialysis unit; RO: Reverse Osmosis; n.a.: non-applicable. (DOCX) [file pone.0247450.s002.docx]

**S2 Table. List of collected variables**

| **Variable** | **Resource use source** | **Unit cost source** |
| --- | --- | --- |
| Hardness | HCB | n.a. |
| Chlorine | HCB | n.a. |
| Conductivity Meter (Water Inlet) | HCB | n.a. |
| Conductivity Master (after 1^st^ RO) | HCB | n.a. |
| Conductivity Slave (after 2^nd^ RO) | HCB | n.a. |
| Colony Forming Units | HCB | n.a. |
| Endotoxin Units | HCB | n.a. |
| Chemical Elements | HCB | n.a. |
| Network Fail | FMC | n.a. |
| Warning | FMC | n.a. |
| Alert | FMC | n.a. |
| Main TO Attendance | HCB | FMC |
| Saturday 1 TO Attendance | HCB | HCB |
| Saturday 2 TO Attendance | HCB | HCB |
| Holiday TO Attendance | HCB | FMC |
| Travel Time | HCB | FMC |
| Routine Time | HCB | FMC |
| Supplementary Task Total Time | HCB | FMC |
| Data Management Time | HCB | FMC |
| Daily Total Time | HCB | FMC |
| Monthly Meeting Time | HCB | HCB |
| Culture Number | HCB | HCB |
| Reagent Hardness 1 | HCB | FMC |
| Reagent Hardness 2 | HCB | FMC |
| Reagent Chlorine | HCB | FMC |
| Total Salt Consumption | HCB | HCB |
| Supplementary Task Total Number | FMC | FMC |
| Filter 20µ (supplementary) | FMC | FMC |
| Filter 10µ (supplementary) | FMC | FMC |
| Filter 5µ (supplementary) | FMC | FMC |
| Filter 1µ (supplementary) | FMC | FMC |
| Resin (supplementary) | FMC | FMC |
| Charcoal (supplementary) | FMC | FMC |
| Reagent Hardness 1 (supplementary) | FMC | FMC |
| Reagent Hardness 2 (supplementary) | FMC | FMC |
| Reagent Chlorine (supplementary) | FMC | FMC |
| Electrolyte Sensor (supplementary) | FMC | FMC |
| Membrane RO (supplementary) | FMC | FMC |
| Pump RO (supplementary) | FMC | FMC |
| Chlorine Sensor (supplementary) | FMC | FMC |
| Chlorine Sensor Gel (supplementary) | FMC | FMC |
| Permeate Conductivity Cell (supplementary) | FMC | FMC |
| Permeate Temperature Cell (supplementary) | FMC | FMC |
| Feed Conductivity Cell (supplementary) | FMC | FMC |
| Feed Temperature Cell (supplementary) | FMC | FMC |
| Concentrate Pressure Sensor (supplementary) | FMC | FMC |
| Storage Battery (supplementary) | FMC | FMC |
| Water Prefine Filter (supplementary) | FMC | FMC |
| Flow Sensor (supplementary) | FMC | FMC |
| T Hout Sensor (supplementary) | FMC | FMC |
| T Hin Sensor (supplementary) | FMC | FMC |
| P 5f Sensor (supplementary) | FMC | FMC |
| Water Consumption | HCB | HCB |
| Electric Consumption (Water Plant) | HCB | HCB |
| Electric Consumption (DU) | HCB | HCB |
| Water Plant Disinfection Time | FMC | FMC |
| Water Plant Disinfection Material | FMC | FMC |
| Revalidation Time | HCB | HCB |
| Filter 20µ | FMC | FMC |
| Filter 10µ | FMC | FMC |
| Filter 5µ | FMC | FMC |
| Filter 1µ | FMC | FMC |
| Calibration Time | FMC | FMC |
| Reagent Calibration Chlorine 1 | FMC | FMC |
| Reagent Calibration Chlorine 2 | FMC | FMC |
| Reagent Calibration Chlorine 3 | FMC | FMC |
| Reagent Calibration Hardness | FMC | FMC |
| Nº of Monthly Dialysis Treatments | HCB | n.a. |
| Conventional Water Technology | HCB | FMC |
| Maintenance Conventional Water Technology | HCB | FMC |
| New Water Technology | HCB | FMC |
| Maintenance New Water Technology | HCB | FMC |

HCB: Hospital Clínic de Barcelona; FMC: Fresenius Medical Care; TO: Technical Operator; DU: dialysis unit; RO: Reverse Osmosis; n.a.: non-applicable
