# Supplementary material for: Health Technology Assessment of a new water quality monitoring technology: Impact of automation, digitalization and remoteness in dialysis units
Source: PLoS One. 2021 Feb 25;16(2):e0247450. doi: 10.1371/journal.pone.0247450 (PMC7906308; doi:10.1371/journal.pone.0247450)
Supplement: S1 Survey — (DOCX) [file pone.0247450.s003.docx]

**S1 Survey**

**Comparative questions**

In this section, there are questions about different aspects of the New Water Technology (NWT) in relation with the Conventional Water Technology (CWT) installed in the DIRAC (Dialysis Centre and Applied Research Clinic). The answers have a Likert Scale format in which you have to answer how you value the NWT compared to the CWT.

Taking as reference the CWT, answer the questions for the NWT:

1. How satisfied are you globally speaking?

| **Much worse** | **Worse** | **Equal** | **Better** | **Much better** |
| --- | --- | --- | --- | --- |
|  |  |  |  |  |

1. How satisfied are you with the organizational process regarding the audits for quality control?

| **Much worse** | **Worse** | **Equal** | **Better** | **Much better** |
| --- | --- | --- | --- | --- |
|  |  |  |  |  |

1. How satisfied are you with the continuous data monitoring for the quality control of the water?

| **Much worse** | **Worse** | **Equal** | **Better** | **Much better** |
| --- | --- | --- | --- | --- |
|  |  |  |  |  |

1. How dynamic are the monthly meetings for the revision of the quality control considering how you receive the information?

| **Much worse** | **Worse** | **Equal** | **Better** | **Much better** |
| --- | --- | --- | --- | --- |
|  |  |  |  |  |

1. How reliable do you feel about the water quality data?

| **Much worse** | **Worse** | **Equal** | **Better** | **Much better** |
| --- | --- | --- | --- | --- |
|  |  |  |  |  |

1. How has your daily workload been modified?

| **Much worse** | **Worse** | **Equal** | **Better** | **Much better** |
| --- | --- | --- | --- | --- |
|  |  |  |  |  |

**Specific questions about the NWT**

In this section there are questions about different aspects of the NWT. There are open questions and suggestion boxes in case you want to give more specific insights that are not collected through the other questions.

1. Sort the following **benefits** from most to least important (being 1 the most important) that you consider the NWT can offer.

| - Usefulness of data available in the tablet (daily and monthly) - Graphically presented data and ease of comprehension - Data reliability - Reduction of time needed to perform preventive/corrective actions - Higher control due to a higher frequency of measurements of the water quality parameters - Others (description and numeric position): ____________________________________ |
| --- |

1. Making a critical appraisal since the NWT was installed, which **benefits** have you encountered with the NWT during this period?

|  |
| --- |

1. Sort the following **problems** from most to least important (being 1 the most important) that you consider the NWT can generate.

| - Potential network fails and no collection/reception of data - Information Technology errors related to the NWT (broken monitor, false warnings/alerts, broken PC hardware, etc.) - Difficulty to understand the data displayed by the new reports - Adaptation/management of the NWT - Others (description and numeric position): ____________________________________ |
| --- |

1. Making a critical appraisal since the NWT was installed, which **problems** have you faced with the NWT during this period?

|  |
| --- |

1. Other comments and suggestions:

|  |
| --- |
