# Supplementary material for: Health Technology Assessment of a new water quality monitoring technology: Impact of automation, digitalization and remoteness in dialysis units
Source: PLoS One. 2021 Feb 25;16(2):e0247450. doi: 10.1371/journal.pone.0247450 (PMC7906308; doi:10.1371/journal.pone.0247450)
Supplement: S1 Fig — (DOCX) [file pone.0247450.s004.docx]

**S1 Fig. Likert scale questions’ results**
